# Kveik Brewing Yeasts Demonstrate Wide Flexibility in Beer Fermentation Temperature Tolerance and Exhibit Enhanced Trehalose Accumulation

**DOI:** 10.3389/fmicb.2022.747546

**Published:** 2022-03-16

**Authors:** Barret Foster, Caroline Tyrawa, Emine Ozsahin, Mark Lubberts, Kristoffer Krogerus, Richard Preiss, George van der Merwe

**Affiliations:** ^1^Department of Molecular and Cellular Biology, University of Guelph, Guelph, ON, Canada; ^2^VTT Technical Research Centre of Finland, Espoo, Finland; ^3^Escarpment Laboratories, Guelph, ON, Canada

**Keywords:** brewing, ale, fermentation, temperature, *Saccharomyces*, kveik, trehalose

## Abstract

Traditional Norwegian Farmhouse ale yeasts, also known as kveik, have captured the attention of the brewing community in recent years. Kveik were recently reported as fast fermenting thermo- and ethanol tolerant yeasts with the capacity to produce a variety of interesting flavor metabolites. They are a genetically distinct group of domesticated beer yeasts of admixed origin with one parent from the “Beer 1” clade and the other unknown. While kveik are known to ferment wort efficiently at warmer temperatures, their range of fermentation temperatures and corresponding fermentation efficiencies, remain uncharacterized. In addition, the characteristics responsible for their increased thermotolerance remain largely unknown. Here we demonstrate variation in kveik strains at a wide range of fermentation temperatures and show not all kveik strains are equal in fermentation performance and stress tolerance. Furthermore, we uncovered an increased capacity of kveik strains to accumulate intracellular trehalose, which likely contributes to their increased thermo- and ethanol tolerances. Taken together our results present a clearer picture of the future opportunities presented by Norwegian kveik yeasts and offer further insight into their applications in brewing.

## Introduction

Traditional Norwegian farmhouse ale yeasts, known as kveik, have captured the attention of the brewing community due to their variation from commonly used brewing yeasts (Norland, 1969; Garshol, 2014; Preiss et al., 2018). These poorly characterized yeasts have been used for centuries in western Norway for traditional farmhouse ale brewing characterized by pitching yeast into wort at temperatures in the 28–40°C range and a consequent fast fermentation completing within 1–2 days (Garshol, 2014). The genome sequences of six kveik strains identified them as having an admixed origin, with one parent strain originating from the “Beer 1” clade, as defined by Gallone et al. (2016), and another as of yet unknown parent (Preiss et al., 2018). Genetic and phenotypic characterizations (Preiss et al., 2018) revealed common signs of domestication and promising beer production attributes [reviewed in Gallone et al. (2018)]. These included efficient flocculation (>80% of strains analyzed) supported by increased copy number variations (CNVs) of various *FLO* genes, efficient consumption of major wort sugars with increased CNVs of maltose and maltotriose metabolic genes (*MAL*), and homozygous loss-of-function single nucleotide polymorphisms (SNPs) in the genes (*PDC1* and *FDC1*) responsible for producing the phenolic off flavor 4-vinylguaiacol, thereby rendering them phenolic off flavor (POF) negative (Preiss et al., 2018). Finally, phenotypic testing showed remarkable ethanol and thermotolerances for kveik strains, thereby broadening the potential application of these yeasts. It is also clear from Preiss et al. (2018) that these kveik strains, while closely related phylogenetically, are genetically and functionally distinct from each other.

The abovementioned fermentation analyses were performed at 30°C. There is currently no further insight into the range of fermentation temperatures at which diverse kveik strains effectively perform fermentations. The reported ethanol and thermotolerances suggest an increased capacity of kveik strains to combat these stresses during fermentation at higher temperatures, thereby increasing their potential for completing higher-temperature fermentations successfully. Insights into the specific fermentation kinetics of diverse kveik strains at a wider temperature range are currently lacking.

Ethanol and thermal stress tolerances are polygenic traits that rely on multiple pathways to combat these environmental impacts on the yeast (Caspeta and Nielsen, 2015; Voordeckers et al., 2015; Snoek et al., 2016; Huang et al., 2018). High ethanol concentrations and temperatures disrupt cell wall integrity, membrane fluidity and integrity leading to leaking of cellular content into the environment, and denature protein structure to ultimately impact its function. In combination, these challenges disrupt the structural integrity of the cell, negatively impacting its metabolism, leading to a decrease in cellular functionality that can lead to cell death. Yeast cells combat increasing ethanol concentrations or high temperatures with intrinsic stress response mechanisms that include stabilizing cell walls and cell membranes, and increasing the cell’s protein folding capacity (Piper, 1995; Viegas and Sa-Correia, 1997). To this end, molecular chaperones, like the heat shock proteins (Hsp), are induced by environmental stress, including thermal stress, to stabilize protein folding thereby protecting against loss-of-function denaturing (Piper, 1995). Furthermore, the production and accumulation of trehalose, a glucose disaccharide that can function as a reserved carbohydrate produced when glucose becomes limiting, is induced by high temperatures and is known to protect the plasma membrane and proteins from environmental (heat, cold, ethanol) and cellular stresses (ROS/oxidative stress) (Coutinho et al., 1988; Singer and Lindquist, 1998; Da Costa Morato Nery et al., 2008; Eleutherio et al., 2015). The intracellular levels of trehalose are controlled by the coordinated regulation of trehalose biosynthetic and hydrolytic reactions, which are regulated by nutrient signaling and stress response pathways during the life cycle (Singer and Lindquist, 1998; Eleutherio et al., 2015). In response to stress, trehalose biosynthesis is induced and hydrolysis by trehalases are reduced to accumulate trehalose intracellularly, where it is proposed to bind and stabilize proteins and membranes (Eleutherio et al., 2015; Magalhaes et al., 2018). A high-affinity trehalose transporter, Agt1, exports accumulated trehalose to the periplasmic space, where it is proposed to bind the polar heads of phospholipids in the outer leaflet of the plasma membrane to protect it from environmental stresses (Eleutherio et al., 1995, 2015; Magalhaes et al., 2018).

To gain further insight into the fermentation characteristics of kveik strains at a range of different temperatures, we compared the fermentation kinetics and sugar metabolisms of six previously characterized kveik strains (Preiss et al., 2018) to commonly used ale yeasts from Beer 1 and Beer 2 clade yeasts (Gallone et al., 2016). In addition, we investigated the potential mechanism(s) that could contribute to the temperature tolerances of these kveik strains.

## Materials and Methods

### Yeast Strains

**FIGURE 1:**
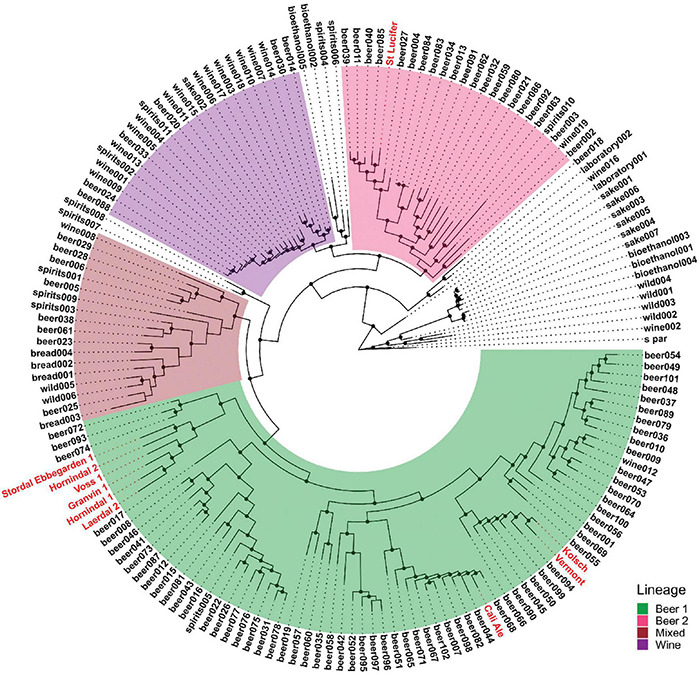
Phylogeny of the strains used compared to the *S. cerevisiae* strains sequenced in Gallone et al. (2016). The four “main” lineages from Gallone et al. (2016) are color-coded and the strains used in this study are shown in red.

We used six previously characterized kveik strains (Preiss et al., 2018) as well as four commonly used commercial ale yeast strains from the Beer 1 and Beer 2 beer yeast families in this study (Table 1). Cali Ale, Vermont Ale and Kölsch are popular, commonly used *Saccharomyces cerevisiae* ale strains of the Beer 1 family (Figure 1; Gallone et al., 2016), which are hypothesized to share a common ancestor with kveik strains (Preiss et al., 2018). St. Lucifer, a Belgian ale yeast of the Beer 2 family (Figure 1), possesses characteristics that are commonly associated with kveik strains, including a high degree of thermotolerance and greater production of fruity flavor compounds but is notably POF + (Gallone et al., 2016). The kveik strains were previously isolated from batch cultures (Preiss et al., 2018). Phylogenetically Hornindal1 and Laerdal2 are closer to each other and Hornindal2 to Ebbegarden1 (Figure 1). All beer yeast strains were supplied by Escarpment Laboratories (Guelph, Ontario). The commercially available Finnish baking and standard sahti brewing yeast Suomen Hiiva (Katina et al., 2007; Catallo et al., 2020; Magalhães et al., 2021) was used as a control in the trehalose and trehalase experiments.

**TABLE 1:** Beer yeast strains used in this study.

Strain name	Source	References
Cali Ale (Beer 1 control)	Escarpment Laboratories	Preiss et al., 2018
Vermont Ale (Beer 1 control)	Escarpment Laboratories	Preiss et al., 2018
Kölsch (Beer 1 control)	Escarpment Laboratories	Gallone et al., 2016
St. Lucifer (Beer 2 control)	Escarpment Laboratories	Preiss et al., 2018
Hornindal 1 (kveik)	Escarpment Laboratories	Preiss et al., 2018
Hornindal 2 (kveik)	Escarpment Laboratories	Preiss et al., 2018
Granvin 1 (kveik)	Escarpment Laboratories	Preiss et al., 2018
Stordal Ebbegarden 1 (kveik)	Escarpment Laboratories	Preiss et al., 2018
Laerdal 2 (kveik)	Escarpment Laboratories	Preiss et al., 2018
Voss 1 (kveik)	Escarpment Laboratories	Preiss et al., 2018
Suomen Hiiva	Suomen Hiiva	Katina et al., 2007

### Wort Preparation

Wort used for beer fermentations and yeast propagation was obtained from a commercial brewery, Wellington Brewery (Guelph, ON). The hopped wort was prepared using Canadian 2-row malt to an original gravity of 12.5°Plato (1.045 specific gravity). The wort was sterilized prior to use at 121°C for 20 min and incubated overnight to the desired fermentation or propagation temperature.

### Propagation and Fermentation

Colonies from YPD plates were inoculated into 100 mL of YPD in biological triplicate and grown at 30°C, 170 rpm until cultures reached the pre-diauxic phase of growth. Cell counts were performed using a haemocytometer and cells were transferred into 100 mL of sterilized wort to a targeted cell density of 5 × 10^6^ cells/mL and grown at 30°C, 170 rpm until pre-diauxic phase. These cultures were counted using a haemocytometer and inoculated at a rate of 1.2 × 10^7^ cells/mL into 200 mL of sterilized wort in 250 mL glass bottles fitted with airlocks. These small-scale fermentations were performed in triplicate at two colder temperatures (12 and 15°C), two standard ale fermentation temperatures (22 and 30°C), and four higher temperatures (35 37, 40, and 42°C). Incubations were for 5 days, except for the 12°C fermentations (10 days). The bottles were incubated without shaking to best approximate typical beer fermentation conditions. Fermentation profiles were acquired by collecting samples throughout the fermentation process and analyzing changes in specific gravity (SG) using a DMA35v4 portable densitometer (Anton-Paar).

### Beer Composition Analysis

Throughout the fermentation, samples were collected and filtered with 0.45 μm syringe filters prior to metabolite analysis. The wort sugar, ethanol and glycerol content were measured using HPLC and a refractive index (RI) detector. The samples collected at the indicated times throughout the fermentation were analyzed using an Aminex HPX-87H column, with 5 mM sulfuric acid as the mobile phase, under the following conditions: flow rate of 0.6 mL/min, 620 psi, and 60°C. Each sample contained 400 μL of filtered sample and 50 μL of 6% (w/v) arabinose as the internal standard.

### Analysis of Trehalose Production and Neutral Trehalase Activity

Colonies were inoculated into 100 mL cultures of YPD and incubated at 30°C with shaking at 180 rpm. Cell samples were collected at the mid-exponential phase of growth and then every 2 h until after the diauxic shift to analyze the timing and quantity of trehalose production. Trehalose was extracted from the cells using 0.5 M cold TCA and analyzed *via* HPLC with an Aminex HPX-87H column at 30°C, 700 psi, and a flow rate of 0.4 mL/min. Concentration of trehalose was determined relative to a standard curve of known concentrations and expressed as milligrams of trehalose per gram of dry cell weight (DCW). Each sample contained 400 μL of unfiltered sample and 50 μL of 6% (w/v) arabinose as the internal standard.

Culture conditions for trehalase activity assays were identical to those previously described for the trehalose assays. Cell samples were collected 2 h post maximum trehalose levels, when trehalase activity is predicted to be highest in all strains. The enzyme kinetics of the hydrolysis of trehalose by neutral trehalase was measured using a stopped assay. Trehalase activity assay was adapted from a previously described assay (Pernambuco et al., 1996). Briefly, cell pellets were collected and resuspended in ice-cold 50 mM MES/KOH (pH 7) + 50 μM CaCl_2_ and crude cell extract was prepared using the glass bead lysis method. Overnight dialysis was performed using a 22 μm Cellu Sep H1 Dialysis Membrane and the dialyzed extract was utilized in stopped trehalase activity assays. The quantity of glucose liberated was determined using a Glucose GOD-PAP Kit (Roche/Hitachi). Trehalase activity was standardized to total protein content using the DC protein quantification technique according to the manufacturer’s recommendations (Bio-Rad).

### Whole-Genome Sequencing and Sequence Analyses

The genomes of the six kveik strains, Cali and Vermont were previously sequenced (Preiss et al., 2018), while those of the Kölsch and St. Lucifer control strains were sequenced here. DNA was extracted from Kölsch and St. Lucifer as previously described (Preiss et al., 2018). Whole-genome sequencing was performed by the TCAG sequencing facility at Sick Kids (Toronto, ONT, Canada). Briefly, an Illumina TruSeq TL paired-end 150 bp library was prepared for each strain and sequenced using an Illumina HiSeq X instrument. This data and WGS data previously generated for the kveik, Cali and Vermont strains (Preiss et al., 2018) were further analyzed. FastQC version 0.11.8 (Andrews, 2010) and fastp version 0.20.1 (Chen et al., 2018) were used to quality-analyze the sequencing reads. Low quality reads and nucleotides were trimmed and filtered using fastp. Filtered reads were aligned against the latest *S*. *cerevisiae* version R64-3-1 genomic reference sequence, which is also known as S288C, using BWA version 0.7.17 (Li and Durbin, 2009). PCR duplicates were marked up and the alignments were sorted and indexed *via* sambamba version 0.7.1 (Tarasov et al., 2015). Per base depth were calculated *via* mosdepth version 0.3.0 (Pedersen and Quinlan, 2018) and FreeBayes version 1.2.0 (Garrison and Marth, 2012) was used to perform variant analysis on aligned reads. Variant effect prediction and the gene names were assigned with SnpEff version 5.0e (Cingolani et al., 2012). Copy number variations of genes were estimated based on coverage with Control-FREEC version 11.6 (Boeva et al., 2011). Wilcoxon Rank Sum test (*p* < 0.05) was performed using the significance script provided by Control-FREEC to evaluate significant copy number variations. Allele frequencies and read depth were plotted to predict the ploidy and aneuploidies for each chromosome.

Phylogenetic analysis was performed using consensus genome sequences generated from variant calls with vcftools (Danecek et al., 2011), which were then aligned to the R64-3-1 genome with minimap2 (Li, 2018). Single nucleotide polymorphisms in the whole-genome alignments were called with paftools.js (included within minimap2), and these were filtered and concatenated using bcftools and bedtools (Quinlan and Hall, 2010). The concatenated SNP matrix was converted into a PHYLIP alignment using vcf2phylip for tree inference with FastTree (Price et al., 2010; Ortiz, 2019). Trees were visualized and annotated in R using the ggtree package (Yu et al., 2017).

### Statistical Analysis

All experiments were performed in biological triplicate and the data was processed and visualized using the ggplot2 and pheatmap packages in R v4.1.2^1^. One-way ANOVA with Tukey’s Honest Significant Difference (HSD) *post hoc* test was performed on all data to ascertain significant differences between treatment means using the agricolae package in R v4.1.2. Threshold of statistical significance was set to *p* < 0.05 and compact letter display was used to report the results of statistically significant pairwise comparisons. Means assigned with a common letter are not significantly different by the HSD-test at the 5% level of significance.

## Results

### Kveik Displays a Wide Fermentation Temperature Range and Accelerated Fermentation Rate

**FIGURE 2:**
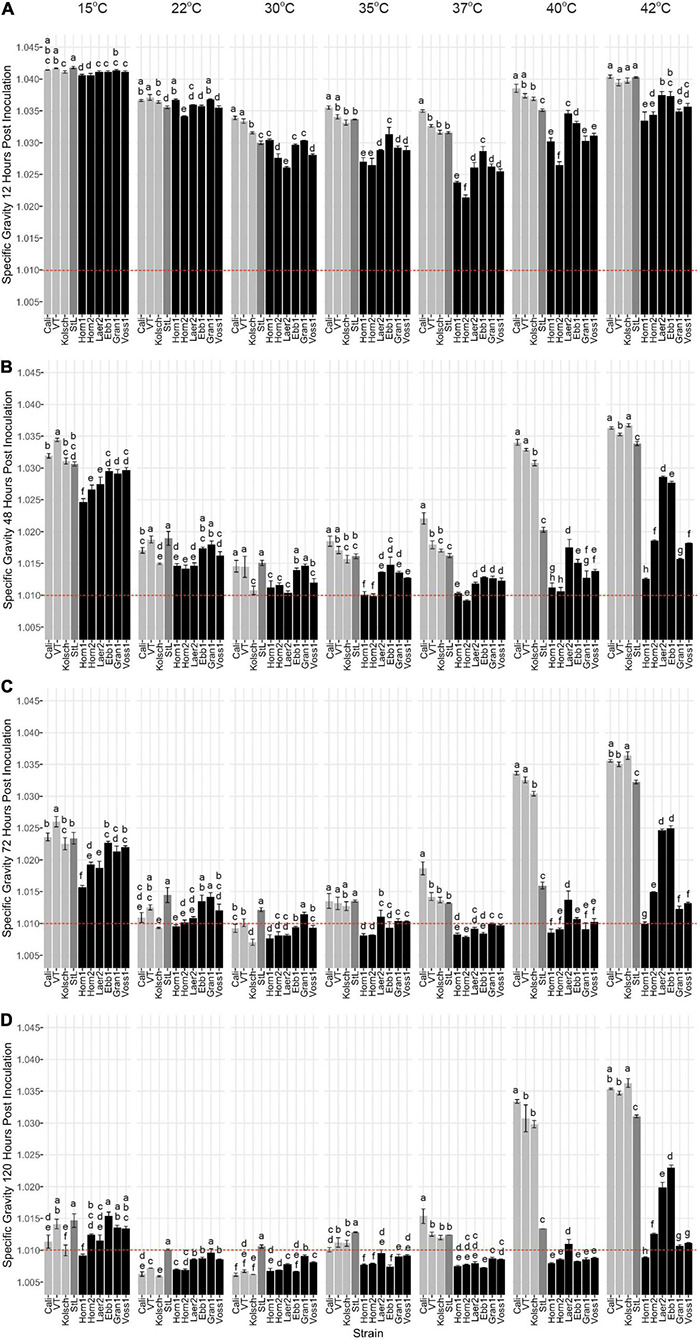
Fermentation profiles comparing four commercial *Saccharomyces cerevisiae* ale strains and six Norwegian kveik isolates at each of the indicated temperatures between 15 and 42°C. Strains were pre-cultured and inoculated into wort as described in the Methods. The fermentation profiles were obtained by recording specific gravities throughout fermentations. Shown are specific gravities measured after **(A)** 12 h, **(B)** 48 h, **(C)** 72 h, and **(D)** 120 h of fermentation. Data points represent the mean of biological replicates (*n* = 3) and error bars represent the standard deviation. Data was subjected to one-way ANOVA followed by Tukey’s HSD analysis of the mean difference in specific gravity between strains for each timepoint and temperature (Specific Gravity ∼ Strain). Mean values assigned with a common letter are not significantly different by the HSD-test at the 5% level of significance within the same temperature. Light gray bars are Beer 1, dark gray bars are Beer 2, and black bars are kveik strains.

While kveik strains are known to be strong fermenters with increased thermotolerance (Preiss et al., 2018), their fermentation efficiencies at a range of temperatures are poorly characterized. We monitored the specific gravities of small-scale wort fermentations at eight different temperatures (Supplementary Figure 1) to gain insight into the temperature-dependent fermentation characteristics of six kveik strains. Variance analyses of the specific gravity (SG) measurements at specific timepoints during these fermentations clearly distinguished the Beer 1 controls from the kveik strains especially at the higher temperatures (Figure 2). With a final gravity (FG) ≤ 1.01 as the target for ales (Parker, 2008), all the control strains displayed their most efficient attenuation at 22 and 30°C (Figure 2D). The Beer 1 control strains reached SG < 1.01 after only 3 days supporting moderate temperatures as optimal for fermentation (Figure 2C), St. Lucifer (Beer 2) never reached an FG < 1.01, regardless of temperature.

**FIGURE 3:**
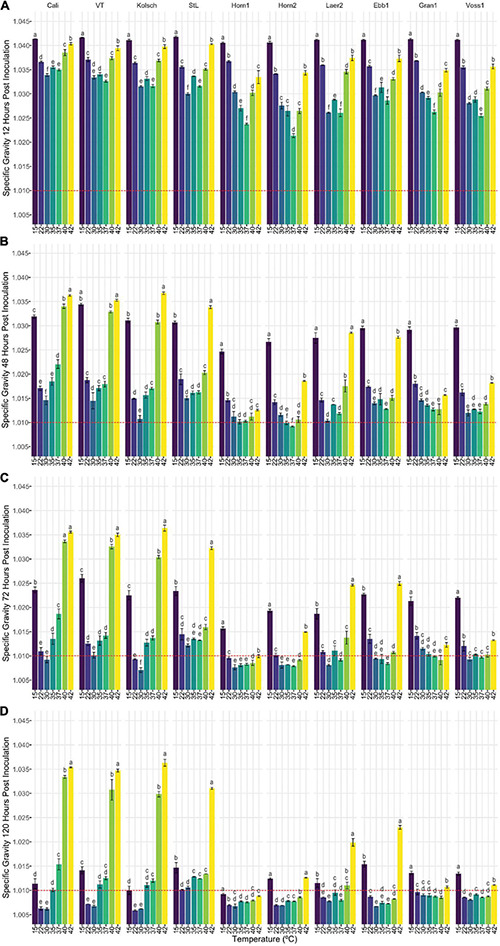
The attenuation of kveik strains vary in a temperature-dependent manner. The data generated in Figure 2 were reorganized to present the temperature-dependent fermentation efficiencies by each of the indicated strains. Shown are specific gravities measured after **(A)** 12 h, **(B)** 48 h, **(C)** 72 h, and **(D)** 120 h of fermentation. Data points represent the mean of biological replicates (*n* = 3) and error bars represent the standard deviation. Data was subjected to one-way ANOVA followed by Tukey’s HSD analysis of the mean specific gravity between temperatures for each timepoint and strain (Specific Gravity ∼ Temperature). Mean values assigned with a common letter are not significantly different by the HSD-test at the 5% level of significance within the same strain.

As most traditional kveik fermentations occur at ∼30°C or higher (Garshol, 2014), we anticipated their optimal fermentation efficiencies would be at higher temperatures. 5/6 kveik strains reached a SG < 1.01 within 5 days at fermentation temperatures between 22–40°C (Figure 2D). In most instances, a SG < 1.01 was already achieved within 3 days at temperatures between 30–40°C (Figure 2C), with Hornindal1 (35°C; 37°C), Hornindal2 (35°C; 37°C) and Laerdal2 (30°C) doing so in 2 days (Figure 2B). The kveiks initiated fermentation faster and notably so at higher temperatures; 5/6 kveik strains achieved ∼30% or more attenuation within 12 h at 30–40°C, while the control fermentations were still lagging (Figure 2A). These accelerated attenuation rates were sustained throughout the fermentation (Figures 2A–C) resulting in FG < 1.01 at 120 h (Figure 2D). Increasing the fermentation temperature to 42°C showed only Hornindal1, Granvin1 and Voss1 approaching FG ∼ 1.01 or lower (Figures 2D, 3). By contrast, Beer 1 control strains displayed increasingly weaker attenuation efficiencies as the temperature increased and essentially stopped fermenting after 48 h at 40 and 42°C, while St. Lucifer’s attenuation rate was only impacted at 42°C (Figures 2C,D, 3).

In addition to strong fermentation capabilities at higher temperatures, our data also demonstrates that some kveik strains possess fermentation efficiencies similar to Beer 1 control strains at lower temperatures. While most kveik strains attenuated similar to the Vermont or St. Lucifer controls at 15°C, Hornindal1 and Laerdal2 completed fermentations similar to the Kölsch and Cali controls, respectively (Figure 2D). Again, some kveik strains and in particular Hornindal1 and Hornindal2, rapidly initiated fermentation at 15°C (Figures 2A–C). In addition, Hornindal1 was the only kveik strain that completed a 12°C fermentation within 10 days along with the Beer 1 controls (Supplementary Figure 3).

Statistical analyses identified variation in the fermentation profiles between kveik strains across all timepoints and temperatures tested. For example, Hornindal1 generally fermented faster and produced beers with the lowest SG, while Laerdal2 and Ebbegarden1 fermented slower to generate beers with higher FG. Furthermore, our analyses of attenuations by individual strains identified distinct temperature-dependent fermentation efficiencies for kveik strains (Figure 3). For example, Hornindal1 shows efficient attenuation at 15–42°C, while Laerdal2 does so at 22–37°C.

### Kveik Exhibit Accelerated Wort Sugar Metabolism

**FIGURE 4:**
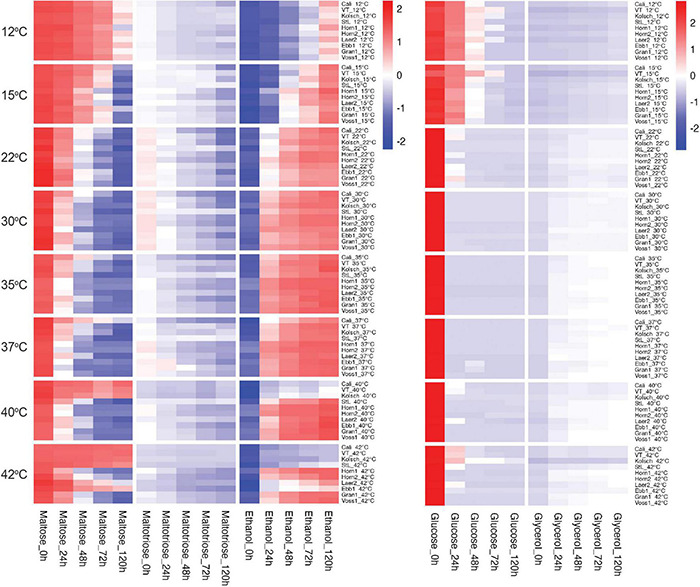
Heatmap of wort sugar consumption and metabolite production of six Norwegian kveik strains and four commercial *Saccharomyces cerevisiae* beer strains during fermentations at a range of temperatures. Samples were collected at the given timepoints and the concentrations of all compounds were determined by HPLC as described in Methods. Data points represent the log1p transformed mean concentrations of biological replicates (*n* = 3).

We next focused on wort sugar metabolism to gain further insight into the accelerated attenuation of the kveik strains. We observed differential sugar consumption, and ethanol and glycerol production rates between strains and temperatures (Figure 4 and Supplementary Figure 2). Sugar consumption (e.g., glucose and maltose) and ethanol production was most efficient at the preferred temperatures and slower metabolism were observed at the less preferred temperatures. Kveik generally performed well in the 30–40°C range, while the Beer 1 controls did so in the 22–30°C range (Figure 4).

**FIGURE 5:**
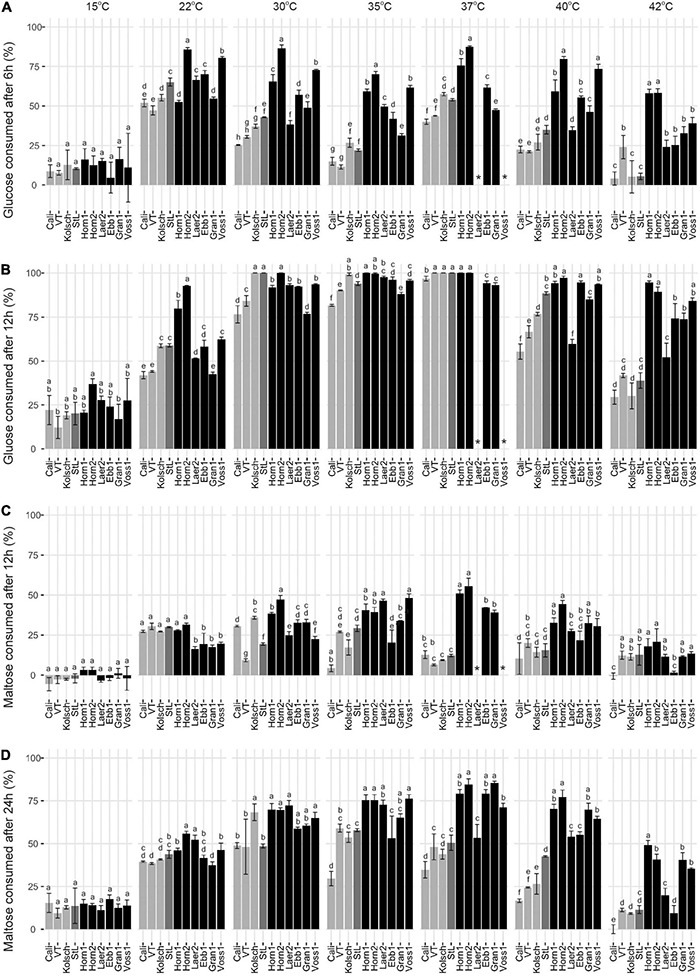
Comparison of the early glucose and maltose consumptions of four commercial *Saccharomyces cerevisiae* ale strains and six Norwegian kveik isolates at each of the indicated temperatures between 15 and 42°C. Samples were collected for HPLC analyses at the same timepoints of SG measurements in Figure 2. The percentage (%) of total sugar consumed post-inoculation are presented for glucose after **(A)** 6 h and **(B)** 12 h, and for maltose after **(C)** 12 h and **(D)** 24 h. Data points represent the mean of biological replicates (*n* = 3) and error bars represent the standard deviation. Data was subjected to one-way ANOVA followed by Tukey’s HSD analysis of the mean sugar consumption between strains for each timepoint and temperature (Concentration ∼ Strain). Mean values assigned with a common letter are not significantly different by the HSD-test at the 5% level of significance within the same temperature. Light gray bars are Beer 1, dark gray bars are Beer 2, and black bars are kveik strains.

Glucose was rapidly consumed by all strains at their preferred temperature optima with most kveiks noticeably faster (50% after 6 h; >90% after 12 h) than most of the controls (Figures 5A,B and Supplementary Figure 2). Also, most of the kveik strains rapidly depleted most of the glucose at higher temperatures (Figure 5B), while glucose consumption was slower by all strains at cooler temperatures (Figures 5A,B).

**FIGURE 6:**
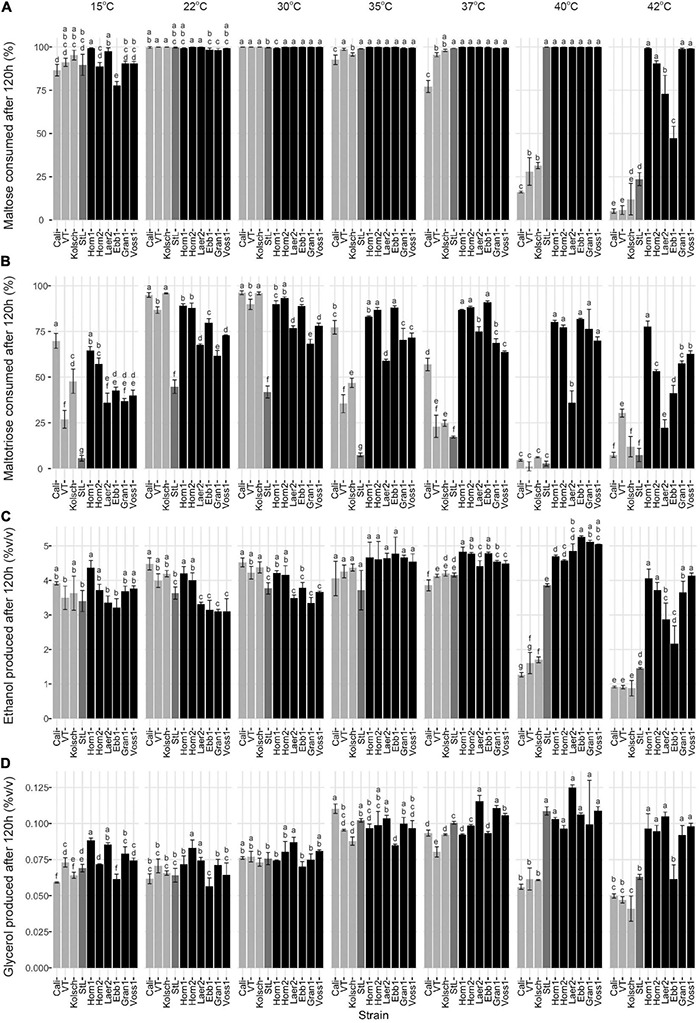
Final wort sugar consumption and ethanol and glycerol production of six Norwegian kveik strains and four commercial *Saccharomyces cerevisiae* beer strains after the fermentation in Figure 2. Samples were collected for HPLC analyses at the same timepoints of SG measurements in Figure 2. The percentage (%) of total sugar consumed after 120 h of fermentation are presented for **(A)** maltose and **(B)** maltotriose, and the amount of **(C)** ethanol, and **(D)** glycerol produced. Data points represent the mean of biological replicates (*n* = 3) and error bars represent the SD. Data was subjected to one-way ANOVA followed by Tukey’s HSD analysis of the mean sugar consumption and metabolite production between strains for each timepoint and temperature (Concentration ∼ Strain). Mean values assigned with a common letter are not significantly different by the HSD-test at the 5% level of significance within the same temperature. Light gray bars are Beer 1, dark gray bars are Beer 2, and black bars are kveik strains.

As maltose is the main sugar in wort, we expected the faster fermentation rates of kveik would be explained by faster maltose consumption. Generally, the control and kveik strains consumed maltose faster at their respective fermentation temperature optima (Figures 5C,D). The kveik strains depleted maltose noticeably faster than the controls with most kveiks consuming at least 30% of this sugar after 12 h and >70% after 24 h of fermentation in the 30–37°C range (Figures 5C,D). However, variability exists in maltose consumption among the kveik yeasts earlier in the fermentation with Laerdal2 and Ebbegarden1 showing slower consumption rates (Supplementary Figure 2 and Figures 5C,D). Finally, all kveik yeasts depleted maltose at 40°C, while Hornindal1, Granvin1 and Voss1 also did so at 42°C (Figure 6A). While St. Lucifer could deplete maltose at 40°C, the Beer 1 controls were deficient in maltose consumption at these higher temperatures.

While kveiks are traditionally used for higher temperature fermentations, we also found most of the kveik yeasts consumed maltose similar to the controls at cooler temperatures after 120 h at 15°C (Figure 6A) and with prolonged incubation (10 days) at 12°C (Supplementary Figure 3). Hornindal1 was notably the most efficient maltose consumer at these colder temperatures (Figure 6A and Supplementary Figure 3).

**FIGURE 7:**
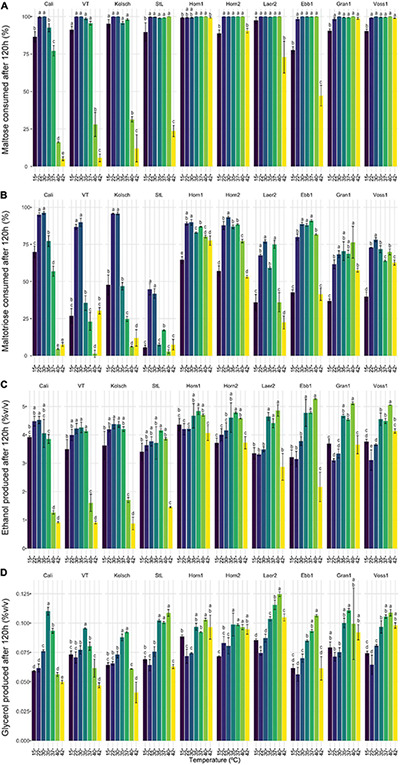
The sugar metabolisms of kveik strains vary in a temperature-dependent manner. The data generated in Figure 6 were reorganized to present the temperature-dependent **(A)** maltose and **(B)** maltotriose consumption, and **(C)** ethanol and **(D)** glycerol production by each of the indicated strains at 120 h of fermentation. Data points represent the mean of biological replicates (*n* = 3) and error bars represent the standard deviation. Data was subjected to one-way ANOVA followed by Tukey’s HSD analysis of the mean sugar consumption and metabolite production between strains for each timepoint and strain (Concentration ∼ Temperature). Mean values assigned with a common letter are not significantly different by the HSD-test at the 5% level of significance within the same strain.

Maltotriose consumption efficiency at a range of temperatures in kveik strains is not known. Here, maltotriose was consumed slower and less completely than the other wort sugars by all strains and fermentation temperature had a pronounced impact. While none of the kveik strains depleted maltotriose completely after 120 h of fermentation (Figures 6B, 7B), Hornindal1 and Hornindal2 initiated maltotriose usage the fastest and at the widest temperature range (30–42°C) (Supplementary Figure 4), and, along with Ebbegarden1 (30–40°C), consumed the most maltotriose (Figures 6B, 7B and Supplementary Figure 4). Statistical analyses confirmed kveik strains have differential abilities to consume maltotriose at their fermentation optima (30–40°C) with Laerdal2, Granvin1 and Voss1 displaying weaker maltotriose consumption (Figure 7B). At 42°C, 4/6 kveik strains consumed >50% of the maltotriose with Hornindal1 being most efficient (Figure 6B). At the colder temperatures, maltotriose consumption was slower. Again, the kveik strains showed some variation in maltotriose consumption at 12°C after a 10-day fermentation with Hornindal1 being the most efficient (Supplementary Figure 3). Thus, while the kveik strains can achieve a FG ∼1.01 after 10 days in a 12°C fermentation, most, with the exception of Hornindal1, will leave a significant amount of maltotriose at the end of the fermentation. Beer 1 control strains consumed maltotriose effectively at 22–30°C, but showed inefficient consumption outside their fermentation temperature optima (Figures 6B, 7B). By contrast, St. Lucifer used ∼25% of the available maltotriose at 22–30°C within the first 12 h and then stalled consumption at all temperatures (Figures 6B, 7B).

The kveik strains produced ethanol and glycerol faster than the controls through the first 24 h of the fermentation, with clear differences present in the 35–40°C temperature range (Supplementary Figure 5). After 120 h all the strains tested displayed similar lower levels of glycerol at the 12–30°C fermentation range, while the control strains, with the exception of St. Lucifer, produced lower levels of glycerol at temperatures beyond 37°C. By contrast, 5/6 kveik strains produced higher levels of glycerol at 35–42°C after 120 h (Figure 6D). The kveik strains produced their highest amounts of ethanol at 35–40°C (Figure 6C). Again, statistical analyses showed temperature-dependent variation in glycerol production by kveik strains. For example, Ebbegarden1 produced increasing amounts of ethanol and glycerol at 35–40°C, but less at 42°C. By contrast, Hornindal1 and Voss1 produced almost double these amounts at 42°C (Figures 6C,D, 7C,D). In combination this data support differential capabilities of the kveik strains at a wide temperature range.

### Kveik Displayed Increased Cell Viability at Higher Temperatures

**FIGURE 8:**
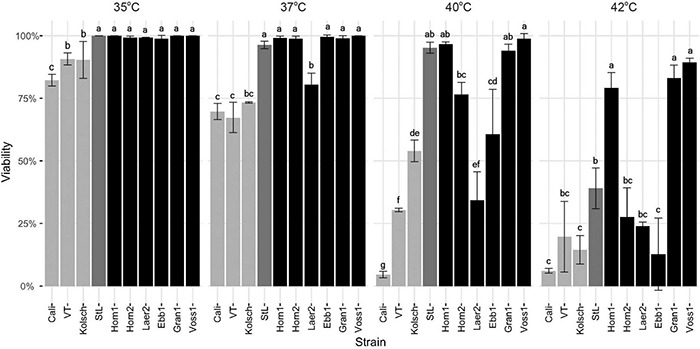
Cell viability following heat treatment. Single colonies of the indicated four commercial ale yeast controls and six Norwegian kveik strains were inoculated into YPD and cultured for 24 h with shaking at 170 rpm at 35, 37, 40, and 42°C. Cell viability was ascertained via staining with Trypan Blue. Error bars represent SD, *n* = 3. Data was subjected to one-way ANOVA followed by Tukey’s HSD analysis of the mean viability between strains for each temperature (Viability ∼ Strain). Mean values assigned with a common letter are not significantly different by the HSD-test at the 5% level of significance within the same temperature.

The kveik strains were clearly metabolically more active while the Beer 1 controls showed significant deficiencies at higher temperatures (Figures 2, 6). As higher temperatures impact yeast cell viability (Lindquist, 1986; Davidson et al., 1996), we measured the survival of these yeasts at 35–42°C. The Beer 1 control strains were impacted at all the temperatures tested as they showed decreased cell viabilities of <75% at 37°C and above, but variable susceptibilities to higher temperatures; e.g., Kölsch showed 50% viability at 40°C, while Cali were <5% viable (Figure 8). St. Lucifer was much more temperature tolerant, only displaying significant cell death at 42°C. By contrast, all the kveik strains, except for Laerdal2, showed no noticeable cell death at 35–37°C. Interestingly, as the temperature increased further, we observed variation in the thermotolerance of kveik strains. The Hornindal1, Granvin1 and Voss1 strains showed more cell survival (>90% at 40°C and >75% at 42°C) than the remaining kveik strains. Hornindal2 still had ∼75% viability at 40°C, but this decreased substantially to only 25% at 42°C (Figure 8). Interestingly, chromosomal copy number analyses revealed the three most tolerant kveik strains had 4n + 1 aneuploidies of Chr IX (Supplementary Table 2). In combination, our data revealed differential tolerances among kveik strains to high temperatures.

### Kveik Exhibit Enhanced Trehalose Accumulation and Reduced Trehalase Activities

**FIGURE 9:**
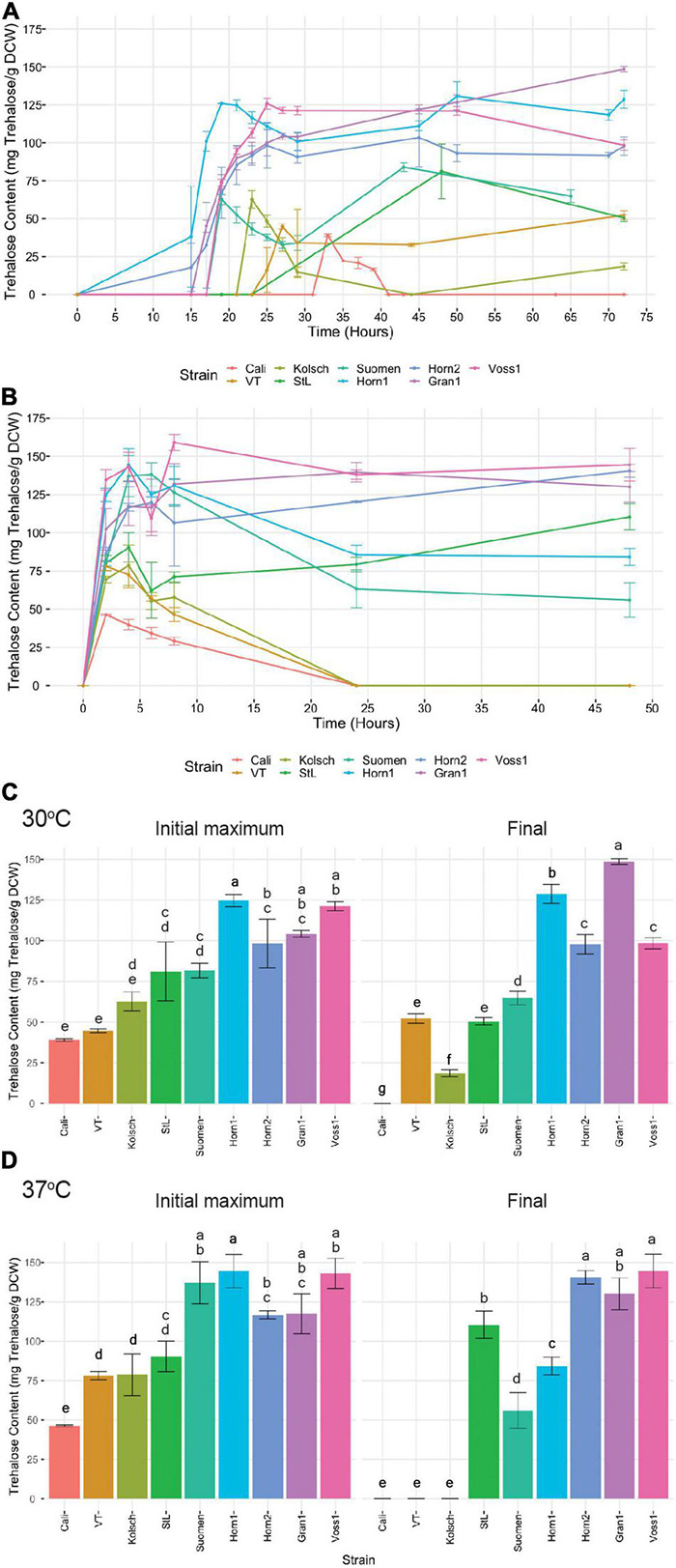
Analysis of trehalose accumulation during growth in YPD at **(A)** 30°C and **(B)** 37°C. Single colonies were inoculated into 100 mL of YPD and incubated at 30 or 37°C with shaking at 170 rpm for 72 and 48 h, respectively. Cell samples were collected at the indicated times and trehalose extractions were performed and concentrations determined *via* HPLC (see “Materials and Methods”). Data points represent the mean of biological replicates (*n* = 3), and error bars represent the standard deviation. The initial maximum trehalose accumulation and final trehalose concentrations were plotted **(C)** for the 30°C and **(D)** for the 37°C growths. Data was subjected to one-way ANOVA followed by Tukey’s HSD analysis of the mean trehalose content between strains for each timepoint and temperature (Trehalose Content ∼ Strain). Mean values assigned with a common letter are not significantly different by the HSD-test at the 5% level of significance.

Trehalose starts to accumulate as a storage carbohydrate in the diauxic phase when glucose becomes depleted as cells transition into stationary phase or is produced as a stress protectant, even in abundant glucose, to combat cold, heat and ethanol stress (Eleutherio et al., 2015). To gain further insight into the tolerance of the kveik strains to the higher fermentation temperatures, we measured trehalose accumulation as a function of time in selected kveik and control yeasts grown at optimal (30°C) and increased (37°C) temperatures. Trehalose was undetectable during the early and mid-exponential phases of growth at 30°C. When cells approached the diauxic shift and most available glucose in the media was depleted, intracellular trehalose rapidly increased (Figure 9A). Not only did the kveik strains produce >1.5-2-fold more trehalose, they also did so much faster than the control beer strains (Figures 9A,C). The kveik strains all produced >90 mg trehalose/g DCW after 22 h of growth, before the control strains produced any detectable trehalose (Figure 9A).

Yeast cells are known to hydrolyse trehalose in stationary phase (Lillie and Pringle, 1980; Parrou et al., 1999). We observed different patterns of trehalose hydrolysis and re-accumulation among the strains analyzed. The control strains displayed a significant hydrolysis of its accumulated trehalose followed by varying efficiencies to re-accumulate trehalose as the cells progressed further into stationary phase (Figures 9A,C). By contrast, the kveik strains consistently maintained high levels of trehalose throughout prolonged growth, but with some variation. While Hornindal1, Hornindal2 and Voss1 showed a reduction of its initial intracellular trehalose shortly after synthesis, trehalose re-accumulation in the Hornindal strains recovered to its original levels during prolonged growth (Figures 9A,C). Interestingly, Granvin1 showed no reduction in intracellular trehalose levels, but strikingly continued to accumulate trehalose throughout post-diauxic growth into stationary phase where it ultimately reached a maximum concentration of 148 mg trehalose/g DCW after 72 h, equivalent to nearly 15% of the dry cell weight. When yeast strains transferred from late exponential phase at 30 to 37°C for further growth, trehalose accumulation peaked within the first 6 h of heat treatment (Figures 9B,C). The Beer 1 control yeasts again accumulated trehalose to lower levels and degraded the accumulated trehalose to levels below detection (Figure 9C). However, St. Lucifer, the more heat tolerant Beer 2 control strain, showed ∼33% reduction in trehalose after 4–6 h of heat exposure, followed by a steady increase in trehalose re-accumulation (Figure 9B). By contrast, the kveik strains and Suomen Hiiva accumulated trehalose within 6 h of the heat treatment to substantially higher levels than the controls (Figures 9B,C). Furthermore, some kveik (Voss1, Granvin1 and Hornindal2) maintained high levels of trehalose, while Hornindal1 and Suomen Hiiva degraded between 40–50% of its accumulated trehalose by 48 h into the heat treatment (Figures 9B,C). In combination, these data show high levels of trehalose accumulation by kveik and the heat tolerant St. Lucifer strains after 48 h at 37°C.

**FIGURE 10:**
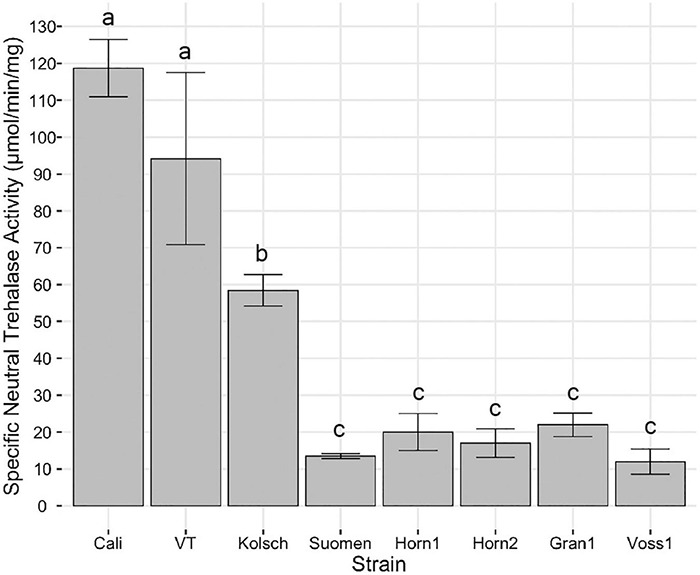
Analysis of specific neutral trehalase activity. Single colonies were inoculated into 100 mL of YPD and incubated at 30°C with shaking at 170 rpm until 2 h post-diauxic shift. Cell samples were collected and trehalase activity assays performed as described in Methods. Specific activity of trehalase is expressed as μmol of glucose liberated per min per mg protein. Data points represent the mean of biological replicates (*n* = 3) and error bars represent the standard deviation. Data was subjected to one-way ANOVA followed by Tukey’s HSD analysis of the mean specific neutral trehalase activity between strains (Trehalase Activity ∼ Strain). Mean values assigned with a common letter are not significantly different by the HSD-test at the 5% level of significance.

Lastly, neutral trehalases are primarily responsible for intracellular trehalose degradation (Nwaka et al., 1995; Elbein et al., 2003; Magalhaes et al., 2018). The consistently higher trehalose accumulation in the kveik strains suggested impaired neutral trehalase activity. We collected samples for trehalase activity assays 2 h after the maximum trehalose spike in post-diauxic growth. The Beer 1 control strains possessed significantly higher neutral trehalase activities (Figure 10). This coincides with the rapid hydrolysis of trehalose after its initial maximum accumulation (Figure 9A). Strikingly, the kveik strains displayed much lower neutral trehalase activities, ranging from 10–18% of the activity supported by Cali (Figure 10).

### Genomic Analyses Reveal Single Nucleotide Polymorphisms in Kveik Trehalose Metabolic Genes

Intracellular trehalose accumulation is controlled by the interplay between its biosynthesis, hydrolysis, and export (Singer and Lindquist, 1998; Eleutherio et al., 2015). All six kveik strains possess SNPs in genes known to be involved with trehalose biosynthesis (Supplementary Table 1). While trehalose biosynthetic genes *TPS1* and *TPS2* both carry SNPs, the regulatory genes *TPS3* and *TSL1* contained more variants. Specifically, *TPS3* have four unique SNPs of which two are present in all strains; 2500A>G (Ser834Gly) is homozygous in all six strains, while 238G>A is homozygous in 3/6 strains (Supplementary Table 1).

Also, the high-affinity trehalose H^+^-symporter Agt1 is important in combatting environmental stress (Da Costa Morato Nery et al., 2008; Ratnakumar et al., 2011) as it is proposed to export intracellularly accumulated trehalose to protect the plasma membrane (Eleutherio et al., 1995; Magalhaes et al., 2018). Our SNP analyses of *AGT1* identified two additional heterozygous frameshift mutations (1175_1176insT in Ebbegarden1 and Laerdal2, and 1772delA in Voss1 and Hornindal2), and gained stop codon mutations [491AAG>TTA (Leu164*) again in Laerdal2 and Ebbegarden1, and 1236C>G (Tyr412*) heterozygous in Hornindal2] (Supplementary Table 1), suggesting heterozygous loss-of-function of *AGT1* in 4/6 kveik strains.

We screened the three yeast trehalases for potential mutations and identified several SNPs in all three genes (Supplementary Table 1). *NTH1* possesses several missense mutations, some homozygous, near the catalytic site in Nth1. In particular, 1213C>T (Leu405Phe) is homozygous in 4/6 kveik strains. *NTH2* carries three unique homozygous SNPs (Supplementary Table 1) in Granvin1, the strain that continuously accumulated trehalose in our assay (Figure 9). Lastly, *ATH1* carries seven unique SNPs, of which three are present in 3/6 or more kveik strains (Supplementary Table 1). These latter three mutations (3394A>G, Asn1132Asp; 3434A>G, Ser1145Asn; 3596A>G; Asn1199Ser) are either homozygous or present in high copy number in Granvin1. In combination, these observations suggest increased trehalose biosynthesis, impaired trehalose export, along with deficient trehalase could lead to the rapid accumulation and maintenance of high levels of intracellular trehalose in kveik.

## Discussion

Kveik are genetically distinct ale yeasts known for rapid fermentation at warmer temperatures (Garshol, 2014; Preiss et al., 2018). Here we provide evidence of strain and temperature-specific variation in fermentation efficiencies of six kveik strains at fermentation temperatures ranging from 12–42°C. These kveik strains also vary in their abilities to survive at higher temperatures. Strikingly, we identified significant increases in intracellular trehalose accumulation in kveik that could contribute to its thermotolerance. Collectively, these findings provide further insight into the benefits and shortcomings of specific kveik strains at a range of different temperatures.

Ale fermentations are usually performed at 15–25°C, with temperatures around 20°C being the norm, while lagers are fermented colder (6–14°C) (Bamforth, 1998). We identify a wide range of efficient fermentation temperatures for kveik strains with a preference for fermentations in the 30–37°C range (Figure 2). Strain variation is an important consideration as some strains, like Hornindal1, can efficiently attenuate, similar to or better than the control strains, at a broad temperature range (15–42°C), while others, like Laerdal2, prefer a narrower temperature range (30–37°C). The kveik strains in general attenuate wort faster than the control strains at their temperature optima (Figure 2). This attribute is appealing as strains like Hornindal1 and Laerdal2 can be used for rapid beer production.

The consumption of wort sugars can further explain kveik’s rapid fermentation. Maltose constitutes ∼60% of the fermentable wort sugar and its metabolism is repressed by glucose (Stewart and Russell, 1998; He et al., 2014). The rapid glucose consumption in kveik could relieve the repression on maltose metabolism, thereby allowing the faster initiation and ultimately complete consumption of maltose at a wider temperature range compared to the control strains (Figures 5–7). The previously identified amplifications of various *MAL* loci in kveik may contribute to its increased maltose consumption (Preiss et al., 2018). However, the efficiency of maltose consumption is strain and temperature dependent. Some strains, like Hornindal1, efficiently consume maltose at all temperatures tested while others, like Ebbegarden1, are less efficient at the temperature extremes tested (Figure 7 and Supplementary Figure 3).

Maltotriose represents ∼20% of the fermentable wort sugar and its incomplete fermentation can be a major concern, especially in high- and very high gravity fermentations. Residual maltotriose causes process inefficiency, a potential undesirable impact on flavor (Pachal and Stewart, 1979) and the added risk of undesirable post-packaging secondary fermentation (Krogerus et al., 2019). The rate limiting step in both maltose and maltotriose consumption is its transport by H^+^-symporters into the cell (Kodama et al., 1995; Stambuk and De Araujo, 2001; Meneses et al., 2002; Rautio and Londesborough, 2003; Alves et al., 2007). Unlike maltose, maltotriose does not have its own high-affinity transporter(s); instead, some maltose transporters can also transport maltotriose, but with much lower affinity. Agt1, the main maltotriose transporter in beer yeast, is a broad substrate range α-glucoside transporter with higher affinity for maltose than maltotriose (Han et al., 1995; Stambuk and De Araujo, 2001; Alves et al., 2007, 2008). Maltotriose transport and its consumption is therefore usually delayed to the latter stages of wort fermentations (Han et al., 1995; Day et al., 2002; Salema-Oom et al., 2005). Nonetheless, increasing the fermentation temperature (15 to 21°C) is known to increase the rate of maltotriose utilization in beer yeasts (Zheng et al., 1994). We found the maltotriose consumption to be both strain and temperature dependent. St. Lucifer is a poor maltotriose consumer as it carries a known frameshift mutation after Glu395 (Glu395fs) in *AGT1*, impacting its ability to efficiently metabolize maltotriose (Gallone et al., 2016). While the Beer 1 and kveik strains showed incomplete maltotriose consumption, the most efficient maltotriose consumption occurred at their respective fermentation temperature optima (Figure 7B). Interestingly, the control strains showed significant reductions in maltotriose consumption at temperatures outside its fermentation optima while not for maltose. Thus, maltotriose consumption is more detrimentally impacted by increased temperatures in the Beer 1 control strains (Figures 6, 7). The kveiks, however, showed more consistent maltotriose consumption across a broader temperature range (Figure 7). Hornindal1, Hornindal2 and Ebbegarden1 were the most efficient maltotriose consumers among the kveiks. Interestingly, the *AGT1* was shown to be non-functional in both lager and Beer 2 yeasts due to a frameshift insertion (Vidgren et al., 2005; Gallone et al., 2016). We identified several heterozygous potential loss-of-function variants of *AGT1* in 4/6 kveik strains (Supplementary Table 1). With premature stop codons previously identified in lager yeasts, the presence of four such loss-of-function mutations could represent altered functionality of Agt1 in kveik yeasts. Furthermore, there are several other SNPs in the kveik *AGT1* genes that were previously identified in lager yeast or that was not reported by Vidgren et al. (2005) (Supplementary Table 1). The impact(s) of these missense mutations on Agt1 function is currently unknown. While these kveik strains phylogenetically form a unique subpopulation of Beer 1 ale yeasts, phased haplotypes previously revealed an admixed ancestry with contributions from outliers in Beer 1 as one haplotype and the other with contributions from diverse origins (Preiss et al., 2018). The origins and larger functional implications for these mutations in *AGT1* in kveik are therefore unknown.

The kveik strains are more thermotolerant than the Beer 1 control yeasts (Figure 8). While the kveik strains generally have increased cell viabilities at higher temperatures, they are not equally thermotolerant as Hornindal1, Granvin1 and Voss1 are clearly more resistant to 40–42°C than Hornindal2, Laerdal2 and Ebbegarden1. The more heat tolerant strains also fermented more efficiently at higher temperatures than the more susceptible strains (Figures 2, 3).

Trehalose is known to protect yeast cells from a variety of environmental impacts, including cold, heat and ethanol stress, by supporting the plasma membrane and suppressing the aggregation of misfolded proteins (Coutinho et al., 1988; De Virgilio et al., 1994; Hottiger et al., 1994; Bandara et al., 2009; Eleutherio et al., 2015). The amount of intracellular trehalose accumulation is determined by its biosynthesis, hydrolysis, and export (Singer and Lindquist, 1998; Eleutherio et al., 2015). Trehalose accumulation under standard growth conditions begins during the diauxic shift when glycogen reserves are being used and continues until stationary phase is reached when its hydrolysis proceeds into the rest of the stationary phase (Lillie and Pringle, 1980; Elbein et al., 2003; Eleutherio et al., 2015). Trehalose biosynthesis is controlled by the Trehalose Synthase Complex (TSC), which is composed of the enzymes Tps1 and Tps2, and two regulatory subunits Tps3 and Tsl1 (Thevelein and Hohmann, 1995; Ferreira et al., 1996; Francois and Parrou, 2001). During stress conditions such as nutrient starvation or exposure to heat (37°C), the TSC is assembled and activated (Francois and Parrou, 2001; Trevisol et al., 2014). The kveik yeasts initiate trehalose accumulation faster and to higher levels than the Beer 1 controls both during prolonged growth at an optimal temperature and induce trehalose production to higher levels in response to heat treatment (Figure 9). All six kveik strains possess SNPs in trehalose biosynthetic genes with *TPS3* carrying potentially impactful loss-of-function homozygous SNPs (Supplementary Table 1). Although a functional link between these SNPs and trehalose biosynthesis have not yet been established, these variants suggest a deregulated TSC could result in the increased trehalose production observed in kveik (Figure 9).

Neutral trehalases Nth1 and the weaker Nth2 function intracellularly to hydrolyze trehalose in the cytosol (Nwaka et al., 1995; Elbein et al., 2003; Magalhaes et al., 2018), while the acidic trehalase Ath1 localizes to the vacuole and is transported to the plasma membrane to hydrolyze extracellular trehalose (Jules et al., 2004, 2008). The activities of the neutral trehalases and Ath1 differ temporally. The Nth1 is active during exponential growth in rich media, which decreases when glucose becomes limiting and is ultimately inactive in starvation (Thevelein, 1984; App and Holzer, 1989; San Miguel and Arguelles, 1994; Thevelein and Hohmann, 1995), while Ath1 is inactive in exponential growth, but becomes active along with Nth2 in stationary phase when the cells starve (Jules et al., 2008; Garre et al., 2009). Strikingly, kveik maintains high levels of intracellular trehalose long after its hydrolysis by the Beer 1 controls during both prolonged growth and in response to heat treatment (Figure 9). The significantly reduced neutral trehalase activities in post-diauxic growth of the kveik strains (Figure 10) could be at least partially responsible for the increased intracellular accumulation of trehalose and hence contributes to stress tolerance. All three trehalase genes in kveik carry several SNPs, some homozygous in several strains, that could impair the functions of the corresponding proteins. Several of these SNPs are homozygous in Granvin1 (Supplementary Table 1), the strain that continuously accumulated trehalose in our assay (Figure 9). It is currently not known how these various SNPs and the coordinated functions of these genes impact trehalose accumulation and ultimately thermotolerance in the kveik strains.

While the molecular basis for the decreased neutral trehalase activity in kveiks remain unresolved, it is known that trehalases are controlled at both the gene transcription and enzyme activity levels in a Protein Kinase A (PKA)-dependent manner (Zähringer et al., 1997; Zahringer et al., 2000). PKA directly phosphorylates and activates Nth1 in exponential growth. Nth1 is active during exponential growth in abundant glucose, it decreases in the diauxic shift and is low in stationary phase cells (Thevelein, 1984; App and Holzer, 1989; San Miguel and Arguelles, 1994; Thevelein and Hohmann, 1995). The activity of Nth1 therefore decreases as glucose becomes limiting as PKA becomes inactive, leading to an increase in trehalose synthesis and accumulation (Schepers et al., 2012; Veisova et al., 2012; Eleutherio et al., 2015). It is currently unclear if the reduction in neutral trehalase activity in kveik is due to a lack of gene expression, reduced protein content, lack of phosphorylation/activation, or inefficient hydrolysis of trehalose due to the SNPs we identified in *NTH1* and *NTH2* (Supplementary Table 1). In addition, the three kveik strains with the highest temperature tolerances, Hornindal1, Voss1 and Granvin1 (Figure 8), also contain a chromosomal aneuploidy of 4n + 1 of chromosome IX (Preiss et al., 2018; Supplementary Table 2). *BCY1*, the gene encoding the regulatory subunit for cAMP-dependent PKA, is located on this chromosome. In the absence of cAMP, Bcy1 inhibits PKA (Hixson and Krebs, 1980; Toda et al., 1987). Overexpression of *BCY1* is linked to reduced PKA activity which leads to a decrease in Nth1 activity and results in greater trehalose accumulation and increased thermotolerance (Portela et al., 2003). Increased expression of *BCY1* in these three kveik strains could contribute to the increased intracellular accumulation of trehalose and reduction in neutral trehalase activity.

While Agt1 is the main maltotriose transporter in beer yeast, it is also a high affinity transporter of trehalose (Han et al., 1995; Stambuk and De Araujo, 2001; Alves et al., 2007, 2008), and is important to combat environmental stress (Da Costa Morato Nery et al., 2008; Ratnakumar et al., 2011). It is proposed to export intracellular trehalose to the periplasmic space where it binds the outer leaflet of the plasma membrane to protect it from environmental stresses (Eleutherio et al., 1995; Magalhaes et al., 2018). We have shown that the strains with the greatest intracellular trehalose content (Hornindal1, Granvin1, and Voss1) maintained significantly higher rates of viability after growth at 42°C (between 80–90% viability) compared to the Beer 1 strains that accumulate less trehalose. Kveik yeasts have also accumulated a variety of SNPs in their *AGT1* genes that have not yet been functionally characterized. While it is currently unknown if any of the *AGT1* mutations impact thermotolerance, it is noteworthy that the strains accumulating loss-of-function *AGT1* SNPs (Ebbegarden1, Laerdal2, and Hornindal2) were more sensitive to heat treatment (Supplementary Table 1 and Figure 5). Agt1 transports trehalose from the environment into the cytosol (Stambuk et al., 1998; Stambuk and De Araujo, 2001; Alves et al., 2008) and also exports trehalose from the cytosol to the external environment (periplasm) (Eleutherio et al., 1995, 2015; Magalhaes et al., 2018). Magalhaes et al. (2018) proposed Agt1 to be a reversible transporter, much like Mal61 for maltose, Fps1 for glycerol and SLC1 for glutamate (Luyten et al., 1995; Van Der Rest et al., 1995; Grewer et al., 2008). In such a scenario, kveik strains could be challenged to export trehalose with high affinity to protect against thermal and ethanol stress for survival, while also importing maltotriose from the environment with low affinity to consume the last available fermentable sugar in the wort. With Agt1 function potentially compromised in kveik strains (and St. Lucifer) through heterozygous loss-of-function mutations, this might explain why these yeasts have residual maltotriose in the beer at the end of the fermentation. Strikingly, the thermosensitive Beer 1 yeasts do not carry the same *AGT1* SNPs as kveik, they do not accumulate intracellular trehalose (Figure 9) and have significant limitations with maltotriose consumption at higher temperatures (Figures 6D, 7D). These hypothesized links between *AGT1* mutation and its potential function(s) during higher temperature fermentations in kveik, needs to be verified experimentally with functional analyses of the various alleles.

Overall, the kveik SNPs we report here provide some insight into the genetic adaptations in genes relevant to various different aspects of fermentation. To fully understand the impacts of these genes on the respective fermentation phenotypes, gene expression analyses and functional assays of the relevant alleles would be needed. Such experiments would be the focus of studies to link these kveik genotypes to a molecular understanding of the respective phenotypes.

Since their introduction to the scientific literature in 2018, traditional Norwegian kveik strains have become extremely popular in the brewing industry, especially in applications with craft breweries and home brewers. The present work demonstrates the variation in kveik strains at a wide range of temperatures and shows that not all kveik strains are equal in terms of fermentation efficiency and stress tolerance. These data can help brewers select optimal temperatures for specific yeast strains to optimize production outcomes. Of relevance to brewers, kveiks are phylogenetically a part of Beer 1 (Preiss et al., 2018) with rapid glucose and maltose metabolisms. However, kveiks share several phenotypic characteristics with Beer 2 yeasts, such as St. Lucifer, including temperature tolerance (Calahan et al., 2011; Tapia et al., 2015). In combination these traits broaden the potential applications of kveik.

This work also presents several opportunities for future development. Here, we showed that kveik is a suitable option for fermentation at high temperatures with potential applications not only for beer production but also in other high-temperature fermentation industries such as distillation or biofuels. We also demonstrate that kveik show enhanced trehalose accumulation, which confers heat tolerance. This is likely linked to polymorphisms in *AGT1*, which may suggest a trade-off between trehalose accumulation and maltotriose consumption. Trehalose accumulation is also known to improve desiccation tolerance, which is a valuable property for active dry yeast (ADY) production. Taken together our results present a clearer picture of the future opportunities presented by Norwegian kveik yeast as well as offer further insight into their applications in brewing.

## Data Availability Statement

The datasets presented in this study can be found in online repositories. The names of the repository/repositories and accession number(s) can be found below: https://www.ncbi.nlm.nih.gov/, PRJNA473622, PRJNA736724.

## Author Contributions

BF, CT, RP, KK, ML, EO, and GM conducted the experiments and data analyses described in this study. KK, ML, and EO performed bioinformatic analysis of the whole genome sequence data. BF, CT, RP, and GM designed the experiments. BF, CT, EO, RP, KK, and GM prepared the manuscript. All authors read and approved the final manuscript.

## Conflict of Interest

RP was employed by Escarpment Laboratories Inc., KK was employed by VTT Technical Research Centre Ltd. The remaining authors declare that the research was conducted in the absence of any commercial or financial relationships that could be construed as a potential conflict of interest.

## Publisher’s Note

All claims expressed in this article are solely those of the authors and do not necessarily represent those of their affiliated organizations, or those of the publisher, the editors and the reviewers. Any product that may be evaluated in this article, or claim that may be made by its manufacturer, is not guaranteed or endorsed by the publisher.

## Footnotes

1
http://www.r-project.org
