# Supplementary material for: Kveik Brewing Yeasts Demonstrate Wide Flexibility in Beer Fermentation Temperature Tolerance and Exhibit Enhanced Trehalose Accumulation
Source: Front Microbiol. 2022 Mar 16;13:747546. doi: 10.3389/fmicb.2022.747546 (PMC8966892; doi:10.3389/fmicb.2022.747546)
Supplement: Supplementary file 3 [file Data_Sheet_1.PDF]

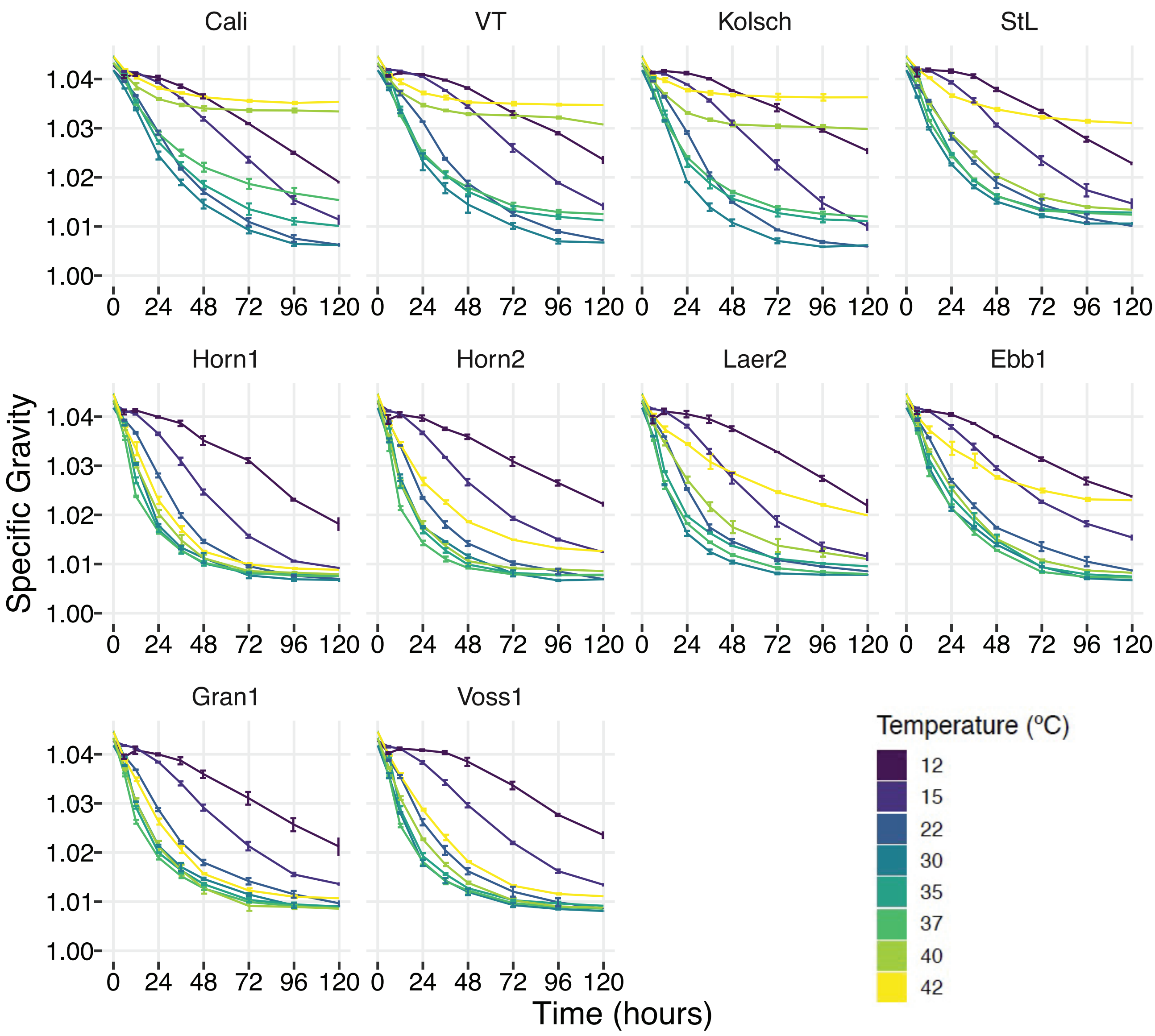

Supplementary Figure S1. Fermentation profiles at the indicated temperatures between 12ºC to 42ºC for four commercial *Saccharomyces cerevisiae* ale strains and six Norwegian kveik isolates. Strains were pre-cultured and inoculated into wort as described in the Methods. The fermentation profiles were obtained by recording specific gravities throughout fermentations. Data points represent the mean of biological replicates (n=3) and error bars represent the standard deviation.
