# Supplementary material for: Kveik Brewing Yeasts Demonstrate Wide Flexibility in Beer Fermentation Temperature Tolerance and Exhibit Enhanced Trehalose Accumulation
Source: Front Microbiol. 2022 Mar 16;13:747546. doi: 10.3389/fmicb.2022.747546 (PMC8966892; doi:10.3389/fmicb.2022.747546)
Supplement: Supplementary file 4 [file Data_Sheet_2.pdf]

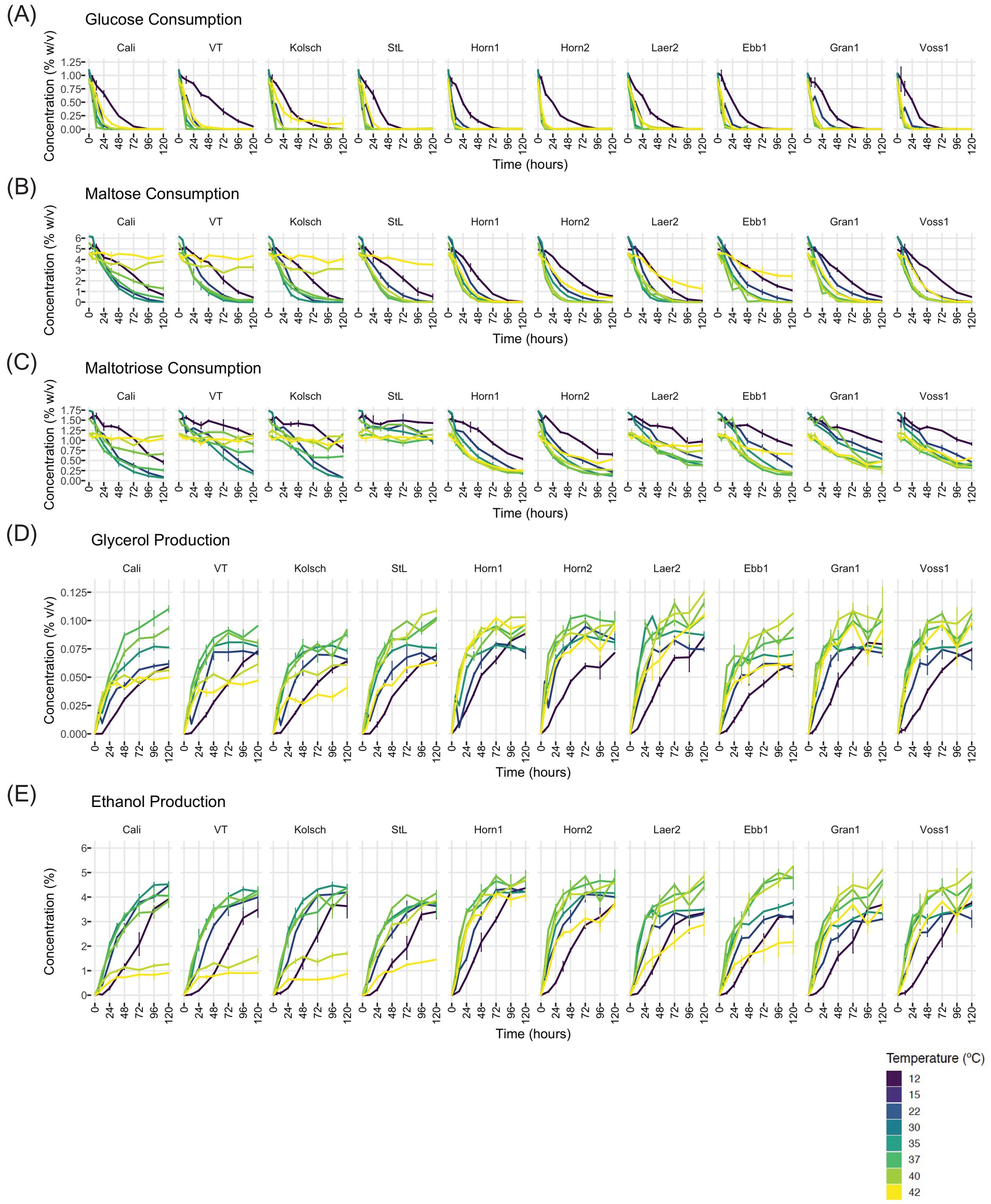

Supplementary Figure S2. Wort sugar consumption and ethanol and glycerol production of six Norwegian kveik strains and four commercial *Saccharomyces cerevisiae* beer strains during the fermentation in Figure 2. Samples were collected for HPLC analyses at the same timepoints of SG measurements in Figure 2. The concentrations of (A) glucose, (B) maltose, (C) maltotriose, (D) glycerol and (E) ethanol were determined by HPLC as described in Methods. Data points represent the mean of biological replicates (n=3) and error bars represent the SD.
