# Supplementary material for: Kveik Brewing Yeasts Demonstrate Wide Flexibility in Beer Fermentation Temperature Tolerance and Exhibit Enhanced Trehalose Accumulation
Source: Front Microbiol. 2022 Mar 16;13:747546. doi: 10.3389/fmicb.2022.747546 (PMC8966892; doi:10.3389/fmicb.2022.747546)
Supplement: Supplementary file 5 [file Data_Sheet_3.PDF]

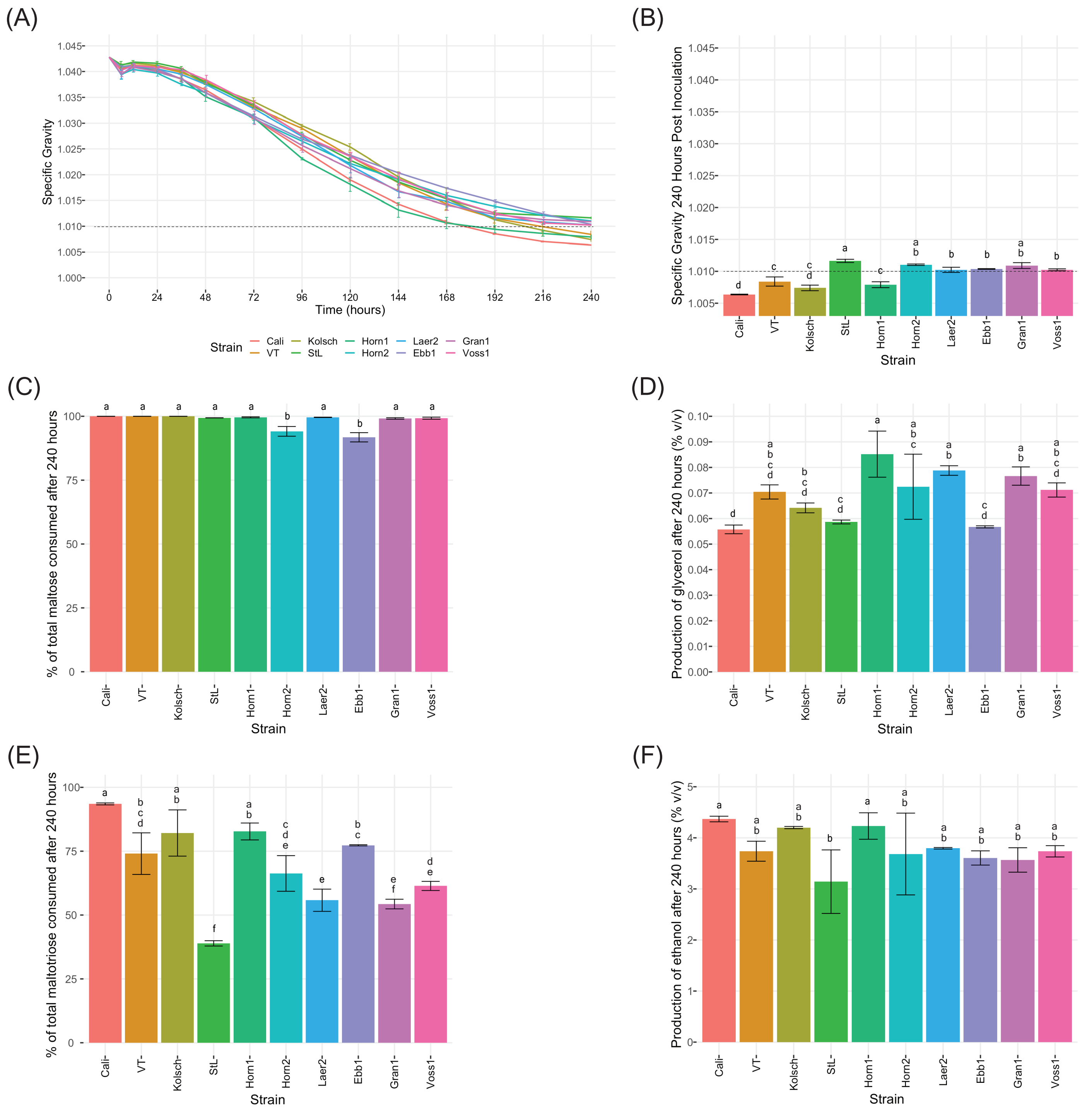

Supplementary Figure S3. Fermentation profiles (A), final gravity at 240 hours (B), percentage wort sugar consumption (C, D), and metabolite production (E, F) of four commercial *Saccharomyces cerevisiae* beer strains and six Norwegian kveik isolates during a prolonged cold fermentation at 12°C for 10 days. Strains were pre-cultured and wort inoculated as described in the Methods. Fermentation profiles were obtained via analyzing the change in specific gravity throughout fermentation using a DMA35v4 portable densitometer (Anton-Paar). Metabolic data was generated through HPLC as described in Methods. Data points represent the mean of biological replicates (n=3) and error bars represent the SD. Data was subjected to one-way ANOVA followed by Tukey's HSD analysis of the mean sugar consumption and metabolite production between strains for each timepoint and temperature (Concentration ~ Strain). Mean values assigned with a common letter are not significantly different by the HSD-test at the 5% level of significance within the same temperature. Light grey bars are Beer 1, dark grey bars are Beer 2, and black bars are kveik strains.
