# Supplementary material for: Kveik Brewing Yeasts Demonstrate Wide Flexibility in Beer Fermentation Temperature Tolerance and Exhibit Enhanced Trehalose Accumulation
Source: Front Microbiol. 2022 Mar 16;13:747546. doi: 10.3389/fmicb.2022.747546 (PMC8966892; doi:10.3389/fmicb.2022.747546)
Supplement: Supplementary file 6 [file Data_Sheet_4.pdf]

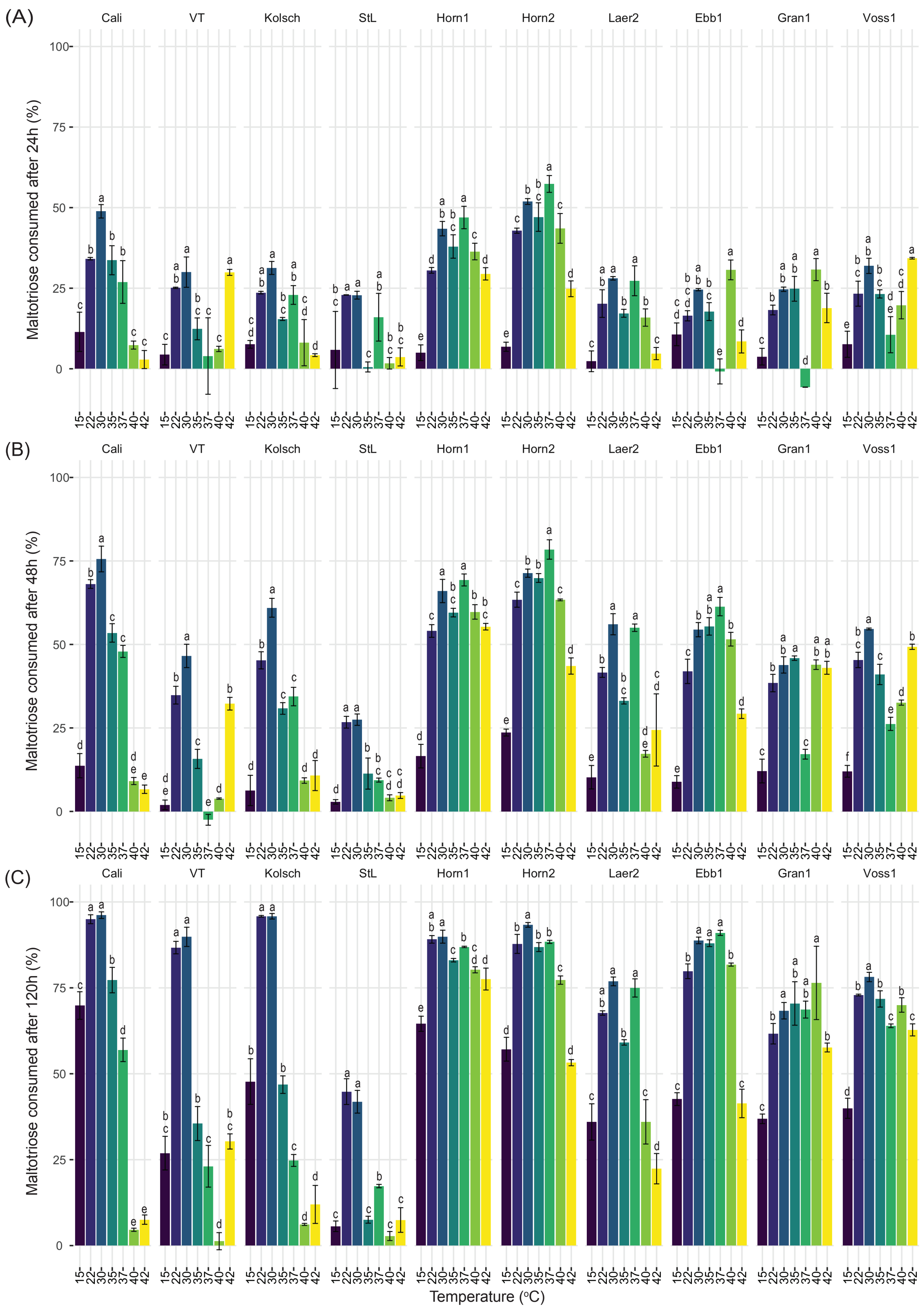

Supplementary Figure S4. Maltotriose consumption of kveik strains during fermentation vary in a temperature-dependent manner. The data generated in Figure 6 were reorganized to present the temperature-dependent maltotriose consumption by each of the indicated strains after (A) 24 hours, (B) 48 hours, and (C) 120 hours of fermentation. Panel (C) is included here again for complete comparison. Data points represent the mean of biological replicates (n=3) and error bars represent the standard deviation. Data was subjected to one-way ANOVA followed by Tukey's HSD analysis of the mean sugar consumption and metabolite production between strains for each timepoint and strain (Concentration ~ Temperature). Mean values assigned with a common letter are not significantly different by the HSD-test at the 5% level of significance within the same strain.
