# Supplementary material for: Kveik Brewing Yeasts Demonstrate Wide Flexibility in Beer Fermentation Temperature Tolerance and Exhibit Enhanced Trehalose Accumulation
Source: Front Microbiol. 2022 Mar 16;13:747546. doi: 10.3389/fmicb.2022.747546 (PMC8966892; doi:10.3389/fmicb.2022.747546)
Supplement: Supplementary file 7 [file Data_Sheet_5.PDF]

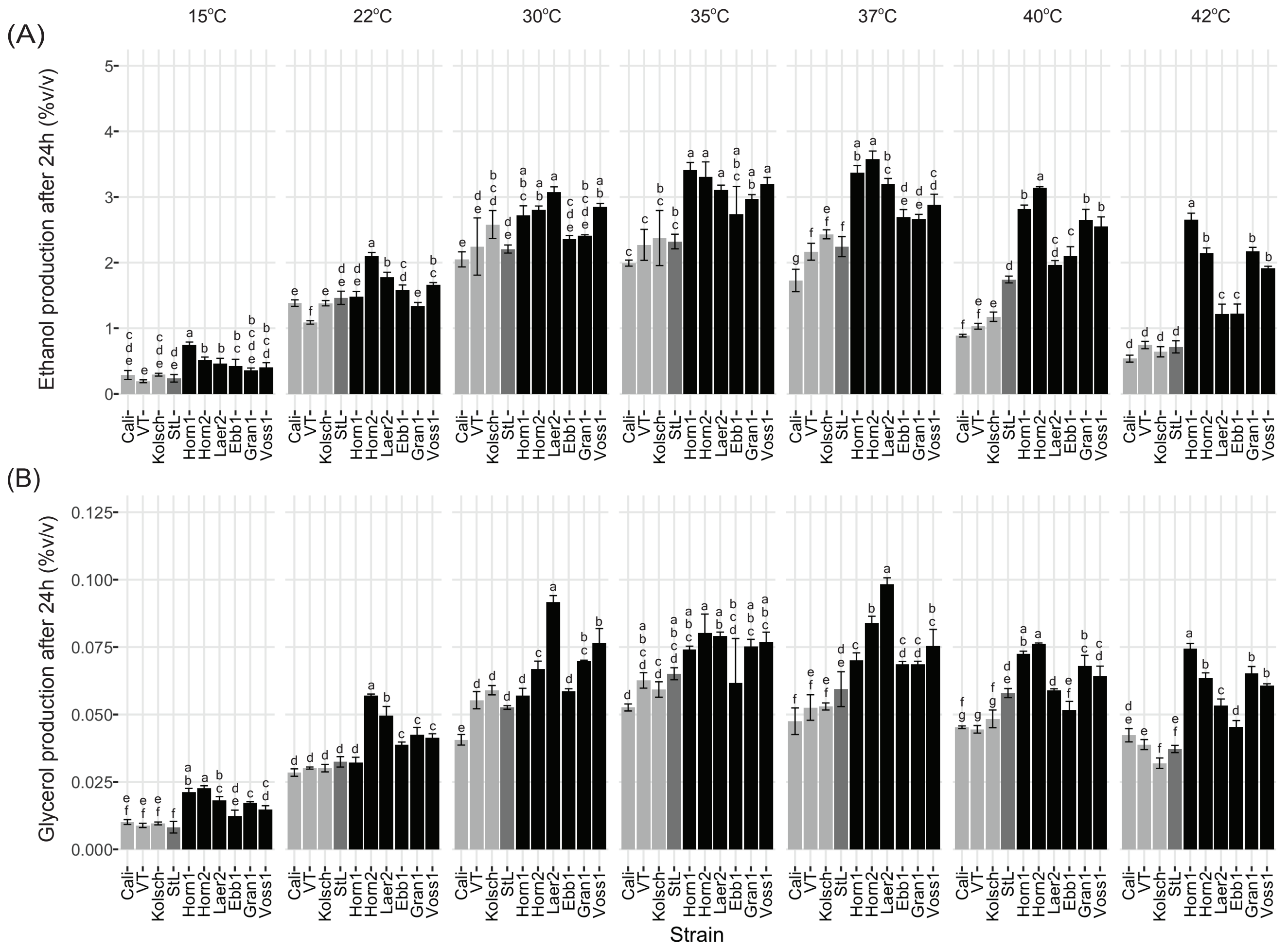

Supplemental Figure S5. Ethanol and glycerol production of six Norwegian kveik strains and four commercial *Saccharomyces cerevisiae* beer strains during the fermentation in Figure 2. Samples were collected for HPLC analyses at the same timepoints of SG measurements in Figure 2. The amounts of (A) ethanol, and (B) glycerol produced after 24 hours of fermenation are presented. Data points represent the mean of biological replicates (n=3) and error bars represent the SD. Data was subjected to one-way ANOVA followed by Tukey's HSD analysis of the mean sugar consumption and metabolite production between strains for each timepoint and temperature (Concentration ~ Strain). Mean values assigned with a common letter are not significantly different by the HSD-test at the 5% level of significance within the same temperature. Light grey bars are Beer 1, dark grey bars are Beer 2, and black bars are kveik strains.
